# The developing world in The New England Journal of Medicine

**DOI:** 10.1186/1744-8603-2-3

**Published:** 2006-03-16

**Authors:** Bernard Lown, Amitava Banerjee

**Affiliations:** 1 Professor Emeritus, Harvard School of Public Health. Senior Physician, Brigham and Women's Hospital. Founder and Chair, ProCOR, Lown Cardiovascular Research Foundation, 21 Longwood Avenue, Brookline, MA 02446 USA; 2 Senior House Officer, General Medicine, John Radcliffe Hospital, Headley Way, Headington, Oxford OX3 9DU, UK

## Abstract

**Background:**

Rampant disease in poor countries impedes development and contributes to growing North-South disparities; however, leading international medical journals underreport on health research priorities for developing countries.

**Methods:**

We examined 416 weekly issues of the New England Journal of Medicine (NEJM) over an eight-year period, January 1997 to December 2004. A total of 8857 articles were reviewed by both authors. The content of each issue was evaluated in six categories: research, review articles, editorial, correspondence, book reviews and miscellaneous. If the title or abstract concerned a topic pertinent to any health issue in the developing world, the article was reviewed.

**Results:**

Over the eight years covered in this study, 1997–2004, in the three essential categories of original research articles, review articles and editorials, less than 3.0 percent of these addressed health issues in the developing world. Publications relevant to DC were largely concerned with HIV and communicable diseases and constituted 135 of the 202 articles of which 63 were devoted to HIV. Only 23 articles addressed non-communicable disease in the DC and only a single article – a book review – discussed heart disease.

**Conclusion:**

The medical information gap between rich and poor countries as judged by publications in the NEJM appears to be larger than the gap in the funding for research. Under-representation of developing world health issues in the medical literature is a global phenomenon. International medical journals cannot rectify global inequities, but they have an important role in educating their constituencies about the global divide.

## Background

The divide between rich and poor countries adversely affects world health, undermines global stability and is one of the challenging problems of the 21^st ^century. Rampant disease in poor countries impedes development and contributes to growing North-South disparities. A stark illustration of this divide is provided by public and private sector expenditures on global health research. Of the $70 billion spent annually, only 10% is allocated to poor countries that bear 90% of the world's disease burden- the so-called "10–90 gap" [1-3]. Similar striking gaps are evident in medical publications. Eight industrialised countries account for nearly 85% of scientific articles, while 163 lower-income countries contribute only 2.5% [4]. Even when the subject matter is closely related to poor countries, their scientists are underrepresented. In 2002, developing world scientists published only 8% of articles appearing in the six major tropical medical journals [3].

Leading international medical journals underreport on health research priorities for developing countries. A significant transatlantic disparity has been noted, with the British Medical Journal (BMJ) and The Lancet providing four times more coverage of diseases affecting primarily poor countries than the New England Journal of Medicine (NEJM) or the Journal of American Medical Association (JAMA) [5].

We examined the NEJM during the past eight years to assess both the magnitude and the trend over this timeframe in covering health issues of developing countries. The Journal was chosen because it is the flagship medical publication in the U.S.A. and contributes significantly to shaping the culture of global medicine. It has been described by its editor as "one of the pre-eminent biomedical publications in the world [6].

## Methods

We examined 416 weekly issues of the New England Journal of Medicine (NEJM) over an eight-year period, January 1997 to December 2004. A total of 8857 articles were reviewed by both authors. The content of each issue was evaluated in six categories: research, review articles, editorial, correspondence, book reviews and miscellaneous. If the title or abstract concerned a topic pertinent to any health issue in the developing world, the article was reviewed.

Designation of a publication as pertaining to developing countries (DC) was determined by four criteria: the material originated in a DC, one of the authors lived in a DC, a disease addressed was largely limited to a poor country, for example HIV/AIDS or malaria, or the topic involved health policies largely pertaining to a DC. Each article designated as pertaining to a DC was expressed as a percentage of the total number appearing in one of the six categories during each successive year.

An article designated as relevant to a DC was then read to determine the factors that may have accounted for its publication. Articles were classified as: (1) HIV-related, (2) Communicable disease (non-HIV), (3) Non-communicable disease, (4) and other. The non-communicable disease articles were too few to analyze into large subcategories such as cardiovascular disease, respiratory disease or cancers. An article related to a DC was included in only a single category.

## Results

Over the eight years covered in this study, 1997–2004, in the three essential categories of original research articles, review articles and editorials, less than 3.0 percent of these addressed health issues in the developing world. (Table) A narrow distribution of content relating to DC was noted among the six categories, ranging from 1.62 percent in correspondence to 3.03 percent for miscellaneous communications. Overall, 202 of the total of 8857 articles published in this period were deemed relevant to a DC (2.3 percent). See Table 1.

**Table 1:** Distribution of 202 articles related to the developing world (DW) in 416 consecutive weekly issues of the NEJM published in 1997–2004

	*Articles*		
	*DW*	*Total*	%

Original research	41	1716	2.39
Reviews	10	511	1.95
Editorials	29	996	2.91
Correspondence	45	2771	1.62
Book Reviews	30	1313	2.28
Miscellaneous	47	1550	3.03
Sum Total	202	8857	2.28

**Figure 1:**
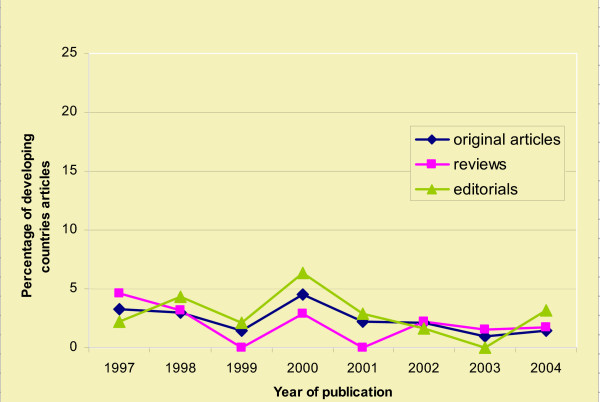
The change in percentage of DC articles; original reviews, editorials, 1997–2004.

Table 2 and Figure 1 demonstrate the trend over the years in the three categories, original research, reviews and editorials, with a low of 1.4% in 1999 to a high in 2000 of 4.8%. See Table 2, and Figure 1.

**Table 2:** The change in percentage of DC articles; original reviews, editorials, 1997–2004 (see figure 1)

**Year**	**1997**	**1998**	**1999**	**2000**	**2001**	**2002**	**2003**	**2004**	**TOTAL**
Original articles-T	209	230	220	220	231	194	202	210	*1716*
Original articles-D	7	7	3	10	5	4	2	3	*41*
**% original article**	**3.3**	**3**	**1.4**	**4.5**	**2.2**	**2.1**	**1**	**1.4**	***2.4***
Reviews-T	65	62	73	69	70	45	68	59	*511*
Reviews-D	3	2	0	2	0	1	1	1	*10*
**% reviews**	**4.6**	**3.2**	**0**	**2.9**	**0**	**2.2**	**1.5**	**1.7**	***2***
Editorials-T	137	141	142	128	138	124	91	95	*996*
Editorials-D	3	6	3	8	4	2	0	3	*29*
**% editorials**	**2.2**	**4.3**	**2.1**	**6.3**	**2.9**	**1.6**	**0**	**3.2**	***2.9***
*Total number of articles*	*411*	*434*	*435*	*417*	*439*	*363*	*361*	*364*	*3224*
*Developing country arti*	*32*	*30*	*12*	*35*	*20*	*19*	*17*	*37*	*202*

T = Total D = Developing countries

For the important category of original articles, the trend appears downward with 3.3% and 3.0% in the first two years of the study to 1.0% and 1.4% in the final two years. A similar trend was noted for review articles.

Publications relevant to DC were largely concerned with HIV and communicable diseases and constituted 135 of the 202 articles of which 63 were devoted to HIV. Remarkably, only 23 articles over the period 1997–2004 addressed non-communicable disease in the DC and only a single article in the 8-year period discussed heart disease and that was in a book review.

## Conclusion

In the years 1997 to 2004 less than 3.0 percent of total publications in the NEJM were devoted to issues relevant to the developing world. Although our study only considered the NEJM, similar findings have been noted in other major USA medical journals [7]. During this period, the global attention of numerous international agencies, nongovernmental organizations and leading industrialized nations has increasingly focused on the plight of poor countries, especially those in sub-Saharan Africa. Much international activity is now directed to reversing the North-South divide, as exemplified by the Group of 8 (G8) nations summit meeting in Gleneagles, Scotland. The summit was intended to set in motion policies to help Africa meet the United Nations Millennium Development Goals to curtail by 2015 poverty and disease among the billions of people who subsist in utter destitution. These activities have not been reflected in the pages of the NEJM.

The medical information gap between rich and poor countries as judged by publications in the NEJM appears to be larger than the gap in the funding for research. Under-representation of developing world health issues in the medical literature is a global phenomenon. Recently Paraje et al [8] reported on health-related publications and their contribution to scientific output. They examined 3.47 million peer-reviewed articles appearing in 4061 journals from 190 countries. Their comprehensive report over a 10-year period, from 1991 to 2002, encompasses an approximately similar time frame as our study. They found that scientists from the 20 most developed economies account for 90 percent of medical publications, while countries with the greatest burdens of disease contribute the least to the scientific health output. Although 23 languages were represented, 96 percent of the articles were published in English. The share of publications from 63 poor countries was less than 2.0 percent of the total. The 46 countries from sub-Saharan Africa published a mere 0.4 percent. These observations have remained largely unaltered over the decade of the study. In fact sub-Saharan Africa declined by about 10 percent while the rest of low-income countries have progressively increased their contributions. Among poorest countries the health information divide is growing.

The paucity of coverage of research from developing countries in Western journals is multifactorial. The World Health Organization has attributed under-reporting to a host of factors including poor research production, faulty manuscript preparation, and lack of access to scientific literature [2]. Research itself is hampered by absence of funding, decrepit laboratory facilities, inadequate training and mentorship opportunities, poorly defined career tracks, weak peer networks, and absence of an organized health-information system. On the other side of the publication divide are constraints on journal editors beholden to the interests of readers who form their subscription base, to advertisers who purchase space to connect with potential customers and to institutions who will buy reprints. Editors also crave an impact factor for the article they publish, namely, to be prominently cited in the scientific press and lay media. None of these powerful incentives focus an editor's attention on poor developing countries.

Richard Horton, editor of The Lancet, has suggested an additional and more disquieting reason for journals failing to reflect the global burden of disease, namely, that, "There is widespread systematic bias in medical literature against disease that dominate the least-developed regions of the world" [3]. He found only 2 participants from low-income countries among 111 editorial board members in five leading medical journals, including the Annals of Internal Medicine, BMJ, JAMA, the Lancet and the NEJM. Each of the five journals Horton examined describes its mission as having global outreach. To justify such a claim, one might anticipate fair and balanced coverage of the prevailing conditions in world health irrespective of disparate economic conditions.

In an increasingly globalized world, leading medical journals have worldwide outreach and impact. The overwhelming majority of reporting on health issues originates in the USA and Western Europe. Skewed coverage of the magnitude and the gravity of global health problems diminishes awareness and impedes mobilization of attention and resources in rich countries to respond to prevailing conditions. Thus inadvertently, publication imbalances adversely affect global health.

While highlighting the underreporting, it is important to note the ongoing significant political tectonic shifts forcing to the fore a new global culture of inclusiveness. The current information revolution promoted by the Internet is educating a growing public on the true state of affairs. This in turn exerts pressure on the political process as exemplified by the G8 conference focusing on debt annulment. Many other significant developments are spurring awareness. Well-endowed departments addressing global health issues have been recently established at Harvard and Yale as well as other leading universities. Several journals have launched initiatives to promote communication of research studies in DC for electronic submission and peer review [9]. Health in the developing world has been a theme recently in issues of the BMJ[10], the Lancet [11], and the Bulletin of the World Health Organization [12]. Science magazine, celebrating its 125^th ^anniversary, is devoting space to a monthly report on scientific research ongoing in a developing region [13]. The editors of JAMA wrote a comprehensive editorial calling for global inclusiveness [14] and devoted an entire issue to the subject [15].

Bridging the communication gap is a two-way street. An infrequent and episodic theme of health in impoverished countries must become a consistent presence. That this is possible is demonstrated by the profound change in coverage of the global burden of risk factors and disease in the two leading UK medical journals. In recent years. the BMJ and the Lancet have materially increased coverage of health issues in the developing world [5].

Medical journals cannot rectify global inequities; nevertheless, they have an important role in educating their constituencies about the global divide. Community responsibility is one of medicine's core values. The lack of visibility of poor countries in international literature is ultimately an issue in medical ethics. The developing world does not exist in isolation [16]; experience of HIV, SARS, avian influenza, growing antimicrobial drug resistance, and terrorism are important reminders that ultimately attention is not dictated by charity but by self-interest. Time and again physicians have been effective agents of change. Attention by medical journals to health conditions of the impoverished world is a wholesome first step.

## Competing interests

The author(s) declare that they have no competing interests.
